# Role of CA125 in predicting ovarian cancer survival - a review of the epidemiological literature

**DOI:** 10.1186/1757-2215-2-13

**Published:** 2009-10-09

**Authors:** Digant Gupta, Christopher G Lis

**Affiliations:** 1 Cancer Treatment Centers of America^® ^at Midwestern Regional Medical Center, Zion, IL, USA

## Abstract

CA125 is the gold standard tumor marker in ovarian cancer. Serum level of CA125 is used to monitor response to chemotherapy, relapse, and disease progression in ovarian cancer patients. Thus, it is reasonable to investigate whether CA125 may have utility as a prognostic indicator as well in ovarian cancer. A large number of epidemiological studies have been carried out to this effect. This review summarizes all available epidemiological literature on the association between CA125 levels and survival in ovarian cancer. To place these studies in context, we provide some background information on CA125 and its role in ovarian cancer.

## Introduction

Ovarian cancer is the leading cause of mortality from gynecologic cancers in the United States (US), resulting in approximately 14,500 deaths annually [1]. The overall lifetime risk of developing ovarian cancer for women in the US is 1.4% to 1.8%. This risk varies from 0.6% for women with no family history, at least three term pregnancies, and four or more years of oral contraceptive use, to 3.4% for nulliparous women with no oral contraceptive use. For women with a family history, the lifetime risk for ovarian cancer is estimated at 9.4% [2]. The overall age-adjusted incidence rate for all ovarian cancer cases as reported by the Surveillance, Epidemiology, and End Results (SEER) Program of the National Cancer Institute is 16.23 cases per 100,000 women standardized to the 2000 US standard population [3]. There is marked geographical variation in age standardized incidence and mortality rates of ovarian cancer, with the highest rates observed in Northern and Western Europe, notably Scandinavia, and in North America [4].

Ovarian cancer is often asymptomatic in its early stages and thus most patients have widespread disease at the time of diagnosis [5]. Unfortunately, the majority of epithelial ovarian cancers remain clinically undetected until patients have developed late stage disease and only a mere 25% of cancers are detected as stage I disease [6]. When diagnosed in Stage I, however, the cure rate can approach 90% with currently available cytoreductive surgery and combination chemotherapy [7]. Ovarian cancer remains a disease that proves fatal to the majority of patients, but where chemotherapy has been established as a treatment that improves survival. A minority of patients attain long survival after such treatment [8]. Despite the achievements of high response rates with surgery followed by chemotherapy [9-11], 75% of women ultimately die of complications associated with disease progression. Once stage III and IV ovarian cancer, which is defined by peritoneal and extra peritoneal metastatic spread, is diagnosed, the survival decreases from 95% at stage I to approximately 20-25% five-year survival despite appropriate treatment [12,13]. Therefore, clinical outcome and possibly survival may be significantly improved by the identification of stage I disease without the need to change surgical or chemotherapeutic approaches [14]. The development of an ovarian cancer-specific biomarker for the early detection of disease has the capacity to improve the dismal survival rate [15].

## Tumor markers in ovarian cancer

The need for the development of reliable serum biomarkers for early detection and prognostication of ovarian cancer, which are both sensitive and specific, remains a long awaited priority. Investigators are aware of this need and the Early Detection Research Network (EDRN) established by the National Cancer Institute has proposed 'guidelines' for the development of screening biomarkers [16]. In the management of ovarian cancer these biomarkers have been applied for monitoring response to treatment, for distinguishing malignant from benign pelvic masses, for estimating prognosis, for predicting response to individual drugs, and for detecting primary disease at an early stage [17]. Several epitopes on the polymorphic epithelial mucin derived from the MUC1 gene have been identified as targets for a family of tumor markers which include CA549, CASA (cancer associated serum antigen), CA19-9, CA15-3, MCA, MOV-1 and TAG72. The cytokeratin proliferation markers TPS and CYFRA21-1 have also been explored in ovarian carcinoma [18]. Amongst these markers the most extensively researched is CA125.

## CA125 in ovarian cancer

The most widely used tumor marker in ovarian cancer, often considered the 'gold standard' is CA125 [19]. It was first identified by Bast, Knapp, and colleagues [20] in 1981. CA125 is a high molecular weight glycoprotein which is raised in approximately 90% of patients with advanced epithelial ovarian cancer [21]. CA125 is expressed by fetal amniotic and coelomic epithelium and in adult tissues derived from the coelomic (mesothelial cells of the pleura, pericardium, and peritoneum) and Mullerian (tubal, endometrial, and endocervical) epithelia. CA125 contains 2 major antigenic domains, namely, A and B, which bind the monoclonal antibodies OC125 and M11, respectively [22]. Since its development, measurement of the serum level of the CA125 antigen has become a standard component of routine management of women with advanced ovarian cancer [23]. CA125 levels of less than 35 U/mL are now accepted as normal [21,24]. When stratified by disease stage, elevated levels were found in more than 90% of patients with advanced stage ovarian cancer but in only 50% of patients with stage I disease [22]. In addition, elevated levels of CA125 are more strongly associated with serous, rather than mucinous tumors [25]. Commonly accepted definitions of disease recurrence based on serum CA125 levels alone specify a doubling of this tumor marker level, either from the upper limit of normal (35 U/mL) in patients with normalization of this marker after primary treatment or from the nadir levels in patients with an elevated serum marker value that never normalizes after primary treatment [26,27].

Numerous studies have confirmed the usefulness of CA125 levels in monitoring the progress of patients with epithelial ovarian cancer [18,28-30]. Most reports indicate that a rise in CA125 levels precedes clinical detection by about 3 months [31]. Unfortunately, a few prospective studies indicated the inadequate sensitivity of CA125 in the setting of ovarian cancer screening in asymptomatic populations [32-34]. Despite the well-characterized limitations in the interpretation of a solitary CA125 value, this biomarker is widely used to prospectively evaluate therapeutic efficacy and monitor disease status among ovarian cancer patients [35,36]. CA125 antigen is a serum marker which has been sufficiently well validated to be of use in routine clinical care [18]. Thus, it is reasonable to seek to determine whether CA125 may have utility as a prognostic indicator and could in the future be used to individualize treatment of patients with ovarian cancer [19]. The goal of this review is to qualitatively summarize the scientific literature on serum CA125 and survival in ovarian cancer and to make recommendations for future research.

## Search Strategy and Selection Criteria

We conducted MEDLINE searches to identify all the studies on the relationship between serum CA125 level and survival in ovarian cancer published between 1985 and 2009. We searched using the terms "survival/mortality/prognosis in ovarian cancer" in combination with the following terms: prechemotherapy serum CA125, postchemotherapy serum CA125, preoperative serum CA125, postoperative serum CA125, CA125 half-life, CA125 nadir level, time to reach nadir level, CA125 Area Under the Curve (AUC). We also searched the bibliographies of the selected papers to identify relevant articles that we might have missed during the primary MEDLINE search. To be included in the review, a study must have: been published in English, reported on data collected in humans with ovarian cancer, had CA125 as one of the predictor variables measured as follows (absolute value, half-life, nadir, time to reach nadir and AUC), had survival as one of the outcome measures (primary or secondary), and had any of the following study designs (case-control, cohort, cross-sectional, prospective, retrospective, case series, longitudinal, clinical trial, meta-analysis). There were no restrictions according to age, ethnicity, type or stage of ovarian cancer. All studies reviewed in this paper have been summarized in tables under separate headings and arranged chronologically by the year of publication.

### Quality Assessment

Although we did not formally rate the quality of reports, we recorded and present information on variables that may reflect the quality of reporting. The variables included study design (retrospective or prospective), years of data collection, sample size, and inclusion of important prognostic factors in multivariate analyses.

## Epidemiologic Studies on CA125 and Ovarian Cancer Survival

### Prechemotherapy Absolute Serum CA125 and Ovarian Cancer Survival

Table 1 summarizes the epidemiologic studies on the association between absolute prechemotherapy CA125 levels and survival in ovarian cancer. A study assessed the relationship between survival and early changes in the serum level of the CA125 antigen in advanced ovarian cancer. While pretreatment CA125 values did not correlate with survival, the concentration of this tumor marker 8 weeks after initiation of therapy was a powerful independent prognostic factor. The median survival for patients (n = 51) with a CA125 < 35 U/ml, vs. patients (n = 50) with a CA125 > 35 U/ml, at this time point, were 26 months and 15 months, respectively. Further, women with serum CA125 values < 50% of their pre-treatment concentration at 8 weeks experienced a median survival of 21 months, compared to only 10 months for individuals with tumor marker levels > 50% of their baseline value [37]. A multicentric study of CA125 kinetics under induction chemotherapy performed in 631 ovarian cancer patients found that prechemotherapy CA125, its half-life, nadir concentration and time to nadir all had a univariate prognostic value for disease free and overall survival [38]. Another prospective study examined the value of pretherapeutic CA125 in 70 consecutive patients with recurrent ovarian cancer before the start of second-line chemotherapy. CA125 was not found to be significantly associated with survival by any of the cutoffs (35, 65, 132, and 339 U/mL) [39].

**Table 1:** Relationship between prechemotherapy absolute serum CA125 levels and survival in ovarian cancer

**First Author, Year, Study Place**	**Data Collection**	**Study Design**	**Sample Size**	**Groups being Compared**	**RR/HR, (95% CI), P-Value**	**Conclusion**	**Variables Adjusted for**
Markman M, 2006, USA, [37]	NA	Longitudinal study	101	≤ 35 U/ml, > 35-- < 100 U/ml, ≥ 100 U/ml	NA	Reduction in serum CA-125 concentration over the initial 2 cycles of chemotherapy was an independent predictor of survival	NA

Riedinger JM, 2006, France [38]	1988 to 1996	Multicentric retrospective study	631	≤ 230 kU/L & > 230 kU/L	Univariate analysis 0.77 (0.73--0.81), < 0.0001	Pre-chemotherapy CA125 had a univariate prognostic value for disease free survival and overall survival	NA

Gronlund B, 2005, Denmark [39]	Dec 1993 to Sep 1998	Prospective study	70	Multiple cutoffs of 35, 65, 132 & 339 U/mL	NA	The pretreatment CA125 level was not found to be significantly associated with survival by any of the cutoffs	FIGO stage, histology, localization of tumor relapse, size of tumor relapse, CASA level

Gadducci A, 1995, Italy [40]	1986 to 1992	Multicentric retrospective study	225	< 500 U/ml, ≥ 500 U/ml	NA	Survival was found to be significantly related to serum CA 125 before the third cycle	FIGO stage, tumor grade, residual disease, CA125 half life

Ron IG, 1994, Israel [41]	Feb 1987 to Dec 1990	Prospective study	48	35-100 U/ml, 101-299 U/ml, 300-499 U/ml, *≥ *500 U/ml	NA, < 0.0001	Early response (CA125 normalcy by the end of the second chemotherapeutic course) was a highly significant predictor of disease-free survival at 12 months	Age, FIGO stage, histology, grade, residual tumor, ascites

Davidson NG, 1991, [42]	Sep 1985 to Sep 1987	Convenience sample	55	< 50 kU/l, 125 ≥ 50 kU/l	NA, < 0.003	Prechemotherapy CA125 level taken 4 weeks after debulking surgery may predict survival in ovarian cancer patients who undergo chemotherapy treatment	Age, Histology, FIGO stage, tumor grade, residual disease

Rustin GJ, 1989, U.K. [43]	April 1985 to Feb 1987	Prospective study	54	≥ 35 U/ml, < 35 U/ml	NA, 0.001	There was a highly significant relationship between the progression free survival time and the change in CA125 levels just prior to chemotherapy	NA

van der Burg ME, 1988, Netherland [44]	Sept 1979 to Dec 1983	Consecutive case series	85	≤ 35 U/ml, 35-60 U/ml, > 60 U/ml	NA	The prechemotherapy level of CA125 on itself is strongly correlated with progression rate and the probability of progression within 3 years	FIGO stage, histology, histological grade, postoperative tumor size

A retrospective multicentric study assessing the prognostic value of the serum CA125 assay in 225 patients with advanced epithelial ovarian cancer found that survival was significantly related to stage, residual disease, tumor grade, serum CA125 before the third cycle, and serum CA125 half-life. Cox proportional hazard model showed that residual disease, serum CA125 half-life, and tumor grade retained a significant value in predicting survival [40]. Another study evaluated the prognostic value of serum CA125 levels both before chemotherapy and after each cycle of one or two courses in 48 patients with advanced ovarian adenocarcinoma. Patients with serum CA125 values below the normal value of 35 U/ml after two courses had a significantly longer median survival and longer disease-free survival than did those patients whose CA125 levels dropped to normal after the third or a later course of chemotherapy [41]. In another study 55 patients with epithelial ovarian carcinoma treated with platinum-based chemotherapy were followed for a minimum period of 2 years. Of these 22 patients had a prechemotherapy serum CA125 level of less than 50 kU/l and 33 patients had a serum CA125 level of greater than or equal to 50 kU/l. The 5-year actuarial survival of the two groups were 75% and 10% respectively [42]. A study evaluating the prognostic value of serum CA125 measurements in 54 patients with advanced ovarian adenocarcinoma found that the change in CA125 levels from before chemotherapy to 1 month later could be used to divide patients into different prognostic groups. The best discrimination was found by dividing the patients into those who showed a greater than sevenfold decrease in CA125 levels and those who showed a smaller change [43]. Finally, a study conducted in 85 patients with epithelial ovarian cancer found that prechemotherapy CA125 level had no prognostic value if the patients were stratified for tumor size [44].

Of the eight studies reviewed under the relationship between prechemotherapy absolute serum CA125 levels and survival, four were prospective, one retrospective, one convenience sample, one consecutive case series type of study. Six studies [37,38,40-43] showed a highly significant relationship between prechemotherapy serum CA125 level changes and survival whereas one study [39] did not find such relationship. In one study, prechemotherapy CA125 was found to be strongly correlated with the probability of progression within 3 years but the data suggesting the relationship between prechemotherapy CA125 levels and survival was not provided [44]. Consequently, the overall data reviewed on the relationship between prechemotherapy serum CA125 levels and survival in ovarian cancer suggests an inverse relationship between the two.

### Postchemotherapy Absolute Serum CA125 and Ovarian Cancer Survival

Table 2 summarizes the epidemiologic studies on the association between absolute postchemotherapy CA125 levels and survival in ovarian cancer. A retrospective study evaluated the prognostic significance of the serum CA125 level after 6 cycles of systemic adjuvant chemotherapy. The median progression-free survival was 26, 14, and 10 months, and the median overall survival was 105, 42, and 37 months in group I (< 10 U/ml), group II (10-21 U/ml), and group III (> 21 U/ml) respectively [45]. One study determined whether CA125 is an independent predictor of overall survival (OS) in patients with surgically defined disease status at the end of primary therapy prior to intraperitoneal (IP) consolidation chemotherapy. When considered as a continuous variable, CA125 was a predictor of OS. Using the median CA125 level as a cut-off, OS was increased in patients with CA125 ≤ 12 U/ml (median 5.8 years) compared with > 12 (3.7 years) [46].

**Table 2:** Relationship between postchemotherapy absolute serum CA125 levels and survival in ovarian cancer

**First Author, Year, Study Place**	**Data Collection**	**Study Design**	**Sample Size**	**Groups being Compared**	**RR/HR, (95% CI), P-Value**	**Conclusion**	**Variables Adjusted for**
Kim HS, 2008, South Korea [45]	Jan 1997 to March 2007	Retrospective study	123	< 10 U/ml, 10--21 U/ml and > 21 U/ml	2.51(1.06-5.92), 0.027 3.13 (1.14-8.61), < 0.001	The serum CA125 level after 6 cycles of primary adjuvant paclitaxel/carboplatin chemotherapy may be a good prognostic factor for survival in complete responders	Residual tumor, chemotherapy cycles

Juretzka MM, 2007, USA [46]	1984 to 1998	Retrospective cohort study	241	CA125 ≤ 12 U/ml vs > 12 U/ml	1.41 (1.05--1.91), 0.0248	CA125 level at the end of primary therapy was a predictor of overall survival and progression free survival	FIGO stage, Histology, grade

Riedinger JM, 2007, France [47]	1988 to 1996	Multicentric retrospective study	494	≤ 35 kU/l, > 35 kU/l	Uni-2.7 (2.2--3.3), < 0.0001 Multi-1.27 (0.94--1.71), NS	CA125 change after first course of chemotherapy was independent prognostic factor for both achievement of pathological complete response and overall survival.	Age, Histology FIGO stage, residual tumor

Badulescu F, 2005, Romania [48]	2000 to 2002	Prospective study	40	NA	NA, < 0.05	The response evaluation criteria based on the blood levels variations of CA125 antigen are a better instrument for the estimation of the compared prognosis with the RECIST criteria	Age, FIGO stage, RECIST criteria

Van Dalen A, 2000, Europe [5]	1994 to 1997	Prospective multicentric study	213	≤ 25 kU/L, > 25 kU/L	5.6 (2.65--11.90), < 0.0001	CA125 level of 25 kU/L on completion of three courses of chemotherapy is a good indicator of 2-year overall survival	FIGO stage, Histology, grade, TPS levels

Ron IG, 1994, Israel [41]	Feb 1987 to Dec 1990	Prospective study	48	≤ 35 U/ml, > 35 U/ml	NA, < 0.0001	Patients with CA125 below 35 U/ml after 2 courses had a significantly longer median and disease-free survival than those whose CA125 dropped to normal after the third or a later cycle	Age, FIGO stage, histology, grade, residual tumor, ascites, prechemotherapy CA125

Fisken J, 1993, UK [49]	NA	Retrospective study	58	4 quartiles < 55 U/ml, 58-221 U/ml, 228-434 U/ml, > 450 U/ml	< 0.0005	CA125 was a highly significant predictor of both progression free and overall survival after the first cycle and throughout primary chemotherapy	Residual disease, age, tumor grade, performance status, ascites

Redman CW, 1990, U.K. [50]	March 1986 to March 1988	Consecutive case series	50	≤ 35 U/ml, > 35 U/ml	NA, 0.0009	Serum CA125 after two courses gave the greatest discrimination between patients alive at 12 months and those who did not survive that long	Age, FIGO stage, histology, grade, residual disease

A retrospective multicentric study was carried out to assess the prognostic value of the CA125 change after the first and the second courses of induction chemotherapy. CA125 change after the first course, residual tumor, CA 125 before the second course and patients' age were independent prognostic factors for OS [47]. A study compared the predicted value of the blood levels variations of CA125 antigen and the imunohistochemical expression of CA125, with imagistic criteria regarding the survival estimation of female patients with relapsed ovarian carcinoma. In multivariate analysis only the variation of blood levels of CA125 and the free disease interval from the finalization of the first line chemotherapy were predictive of survival, while the other variables, including the RECIST criteria, had no impact on survival [48]. Another prospective multicentric study evaluated the prognostic significance of CA125 and TPS levels above the discrimination value (25 kU/L and 100 U/L, respectively). Tumor marker levels in stage I and II were not correlated with survival. However, stage III and IV patients with elevated levels of CA125 or TPS after three chemotherapy courses had a worse 2-year OS (69% vs 26%, and 57% vs 20%, respectively) than patients with normal levels of the markers [5].

One study examined the prognostic value of early serum CA125 assay in 58 patients with advanced epithelial ovarian cancer. CA125 was a highly significant predictor of both progression free and overall survival after the first cycle and throughout primary chemotherapy. Patients in the upper quartile (CA125 > 450 U/ml) had a very poor median survival of 7 months while those in the lower quartile (CA125 < 55 U/ml) had a good median survival of 23 months. Those in the two interquartile groups, who had CA125 levels ranging from 58-221 U/ml and 228-434 U/ml, had relatively intermediate median survival times of 16 months and 15 months respectively [49]. Another study found that patients with serum CA125 values below 35 U/ml after two chemotherapy courses were significantly more likely to achieve complete remission and had a significantly longer median survival. In multivariate analysis, serum CA125 levels after two courses were the most important independent prognostic factor [50].

Out of the eight studies reviewed in this section, three were retrospective, three prospective and one consecutive case series. Of these, seven studies [41,45-50] demonstrated that postchemotherapy serum CA125 level is a good prognostic indicator for survival. These studies suggest that patients with serum CA125 values within the normal range after chemotherapy had a significantly longer overall and disease-free survival than did those patients whose CA125 levels remained high after chemotherapy. In one of the studies CA125 below 25 kU/l after 3 chemotherapy courses was not significantly correlated with overall survival in stage I and II patients, although it was, in the subgroup of patients with stage III and IV disease [5]. Overall, there is a large body of evidence to suggest that postchemotherapy CA125 level is a good predictor of overall and progression free survival in ovarian cancer.

### Absolute Serum CA125 during Chemotherapy and Ovarian Cancer Survival

Table 3 summarizes the epidemiologic studies on the association between absolute CA125 levels during chemotherapy and survival in ovarian cancer. A retrospective study assessed the prognostic value of prechemotherapy serum CA125 level, CA125 kinetics, and CA125 half-life in advanced ovarian cancer during induction cisplatin polychemotherapy. The prechemotherapy CA125 level had no prognostic value for survival. However, the median survival time of patients with CA125 levels below the upper normal limit of normality after two courses of CT was 101 months compared to a median survival of 21 months in patients without CA125 normalization [51]. Another retrospective multicentric study assessed the prognostic value of the serum CA125 assay in 225 patients with advanced epithelial ovarian cancer. Multiple logistic regression showed that residual disease, serum CA 125 half-life, serum CA 125 before the third cycle, and serum CA 125 before the first cycle retained a significant value in predicting second-look findings. Survival was significantly related to stage, residual disease, tumor grade, serum CA125 before the third cycle, and serum CA125 half-life [40]. Another study investigated the serum CA125 regression after cytoreductive surgery and during the first three courses of chemotherapy in 60 ovarian cancer patients. Within stage III-IV patients, a significant positive correlation was seen between survival and (a) stage III, (b) residual tumor ≤ 1 cm, (c) CA125 normalisation after three courses and (d) CA125 half-life ≤ 20 days. The median survival times of patients with and without a CA 125 normalization after three courses were 27 and 14 months respectively [52].

**Table 3:** Relationship between absolute serum CA125 levels during chemotherapy and survival in ovarian cancer

**First Author, Year, Study Place**	**Data Collection**	**Study Design**	**Sample Size**	**Groups being Compared**	**RR/HR, (95% CI), P-Value**	**Conclusion**	**Variables Adjusted for**
Colakovic S, 2000, Yugoslavia [51]	NA	Retrospective study	222	≤ 35 U/ml & > 35 U/ml	< 0.0001	The time needed for normalization of CA125 levels can divide patients into good and poor prognostic groups early during chemotherapy	Therapeutic response Karnofsky index, residual disease, tumor grade, CA125 half-life, CA125 kinetics

Gadducci A, 1995, Italy [40]	1986 to 1992	Multicentric retrospective study	225	< 35 U/ml & > 35 U/ml before the third cycle of chemotherapy	NA	Serum CA125 half-life during early chemotherapy was an independent prognostic factor for both the achievement of a pathological complete response and the survival of patients with advanced epithelial ovarian cancer	FIGO stage, tumor grade, size of residual disease, serum CA125 before the first cycle of chemotherapy & serum CA125 half life

Yedema CA, 1993, Netherlands [52]	July 1984 to Dec 1990	Retrospective study	60	≤ 35 U/ml & > 35 U/ml	5.60 (1.16-27.1), 0.03	There was a significant co relationship between serum CA125 levels after three courses of chemotherapy and survival in ovarian cancer.	Stage, histology, grade, tumor rest

All three studies reviewed above were retrospective. Collectively, the findings from these studies coupled with those reported in tables 1 and 2 provide further evidence supporting the prognostic role of CA125 throughout the entire spectrum of chemotherapy treatment in ovarian cancer.

### Preoperative Absolute Serum CA125 and Ovarian Cancer Survival

Table 4 summarizes the epidemiologic studies on the association between absolute preoperative CA125 levels and survival in ovarian cancer. A retrospective study of 75 patients with epithelial ovarian carcinoma found that the preoperative CA125 levels did not correlate significantly with stage, tumor grade or survival. Reduced survival was noted with increasing age at the time of surgery and bulk of the residual disease postoperatively [53]. Another study evaluating preoperative CA125 as a prognostic factor in stage I epithelial ovarian cancer found that patients with preoperative serum CA125 levels < 65 U/mL had a significantly longer survival compared to stage I EOC patients with preoperative serum CA125 ≥ 65 U/mL [54]. Another study assessing the association of preoperative CA125 levels with outcome found that after adjusting for covariates, there was a significant association between CA125 levels and disease-specific survival. As preoperative CA125 levels increased, the risk of death increased except at the highest values of CA125 [55].

**Table 4:** Relationship between preoperative absolute serum CA125 levels and survival in ovarian cancer

**First Author, Year, Study Place**	**Data Collection**	**Study Design**	**Sample Size**	**Groups being Compared**	**RR/HR, (95% CI), P-Value**	**Conclusion**	**Variables Adjusted for**
Osman N, 2008, Limerick [53]	Jan 2001 to Dec 2005	Retrospective study	75	≤ 500 u/ml, > 500 u/ml	NA, 0.85	The preoperative CA125 level did not correlate significantly with stage, tumor grade or survival	Age, histology, FIGO stage, grade

Petri A, 2006, Denmark [54]	Dec 1994 to May 1999	Retrospective study	118	< 65 U/mL, ≥ 65 U/mL	3.4 (1.2--9.6), 0.01	Patients with stage I EOC and preoperative serum CA125 levels < 65 U/mL had a significantly longer survival compared to those with serum CA125 ≥ 65 U/mL	Age, histology FIGO substage, grade, chemotherapy

Cooper BC, 2002, USA [55]	1990 to 1996	Retrospective study	142	< 160, 160--399, 400--924, 925--2399, 2400 U/mL	GI-Reference, GII-2, GIII-1.5, GIV-4, GV-2, 0.03 (for trend)	There was a significant association between CA125 levels and disease-specific survival	Age, histology FIGO stage, grade, ascites, and optimal cytoreduction

Buller R, 1996, USA [56]	1987 to 1982	Retrospective study	126	≤ 500 U/ml, > 500 U/ml, ≤ 3000 U/ml, > 3000 U/ml	I-HR-NA, 0.48 II-HR-NA, 0.65	Preoperative CA125 levels did not predict survival advantage over a range of cut points (400 to 3000 U/ml)	Age, histology, FIGO stage, tumor grade, residual disease, time to initial chemotherapy

Geisler JP, 1996, USA [57]	NA	Consecutive case series	82	NA	NA, 0.047	In epithelial ovarian carcinoma, high preoperative serum levels of CA125 predict decreased length of survival	Histology FIGO substage, grade

Gadducci A, 1995, Italy [40]	1986 to 1992	Multicentric retrospective study	225	≥ 500 U/ml, < 500 U/ml	NA	Serum CA125 half-life during early chemotherapy was an independent prognostic factor for survival	FIGO stage, tumor grade, size of residual disease & serum CA125 half life

Nagele F, 1995, Austria [58]	Jan 1984 to June 1993	Retrospective study	201	< 65 U/mL, ≥ 65 U/mL	Uni-7.45 (2.83-19.65), < 0.001 Multi-6.37 (2.39-16.97), < 0.001	Preoperative CA125 was the most powerful prognostic factor for survival	Age, FIGO substage, grade,

Makar AP, 1992, Norway [59]	1983 to 1990	Prospective study	200	≤ 150 U/ml, > 150 U/ml	NA, 0.035	Preoperative CA125 did not appear to be of any prognostic value in epithelial ovarian cancer	NA

Sevelda P, 1989, Austria [60]	NA	Prospective study	163	≥ 35 U/ml, < 35 U/ml	NA, 0.13	Preoperative CA125 was not a predictor of survival	Histologic grade, FOGO stage, residual tumor

Moebus V, 1988, Germany [61]	NA	Convenience sample	202	≥ 65 U/ml, < 65 U/ml	NA, 0.005	Significantly longer survival was noted for patients with preoperative values below 65 U/ml than for those with values above 65 U/ml	NA

Cruickshank D, 1987, Aberdeen [62]	NA	Prospective study	52	≥ 35 U/ml, < 35 U/ml	NA	No correlation was found between CA125 concentration and survival	NA

One study determined the importance of the rate of decline of CA125 relative to conventional prognosticators of ovarian cancer survival and found that upon univariate analysis, slope of the CA125 exponential regression curve, number of cycles to normal CA125 levels, residual disease, and platinum treatment intensity were the most important predictors of survival [56]. Another study evaluated the relationship between the degree of elevation of preoperative CA125 and length of survival in ovarian cancer. Decreased length of survival was found to be related to the degree of elevation of CA125 prior to initial exploratory laparotomy. The mean initial CA125 for patients surviving five years or more (15 patients) was 899 U/mL, with an SD of +/- 1,880 U/mL, while the CA125 for patients surviving less than five years (67 patients) was 1,978 U/mL, with an SD of +/- 1,852 U/mL [57].

A study evaluating the prognostic importance of preoperative CA125 in patients with stage I epithelial ovarian cancer found that in univariate analysis, overall survival decreased significantly in patients positive for CA125 (≥ 65 U/mL). Multivariate analysis identified preoperative CA125 as the most powerful prognostic factor for survival, the risk of dying of disease being 6.4 times higher in CA125-positive patients [58]. In a study the prognostic significance of the serum CA125 level was evaluated in 687 patients with invasive epithelial ovarian malignancies. Using Cox multivariate analysis, the preoperative serum CA125 level showed no independent prognostic significance, whereas the postoperative level did [59]. In a study serum CA125 levels determined before surgery and 3 months after surgery were evaluated as independent prognostic factors for survival. CA125 gave no additional information with regard to the relationship of survival prognosis to histologic grade and to the diameter of residual tumor mass [60].

A study evaluated whether the pre- and postoperative determination of CA 125 improves the prognostic information at the time of primary operation. There was a significantly longer survival for patients with preoperative values below 65/U/ml than for patients with levels above 65 U/ml. Elevated postoperative values also resulted in poor prognosis. The study found a survival of 5% after 5 years in this group vs. 42% for patients with normal postoperative values. The best prognosis was found in patients with pre- and postoperative values lower than 65 U/ml [61]. In a prospective study of 52 patients with ovarian malignancy followed up for 3-18 months the clinical significance of pre-operative serum CA125 as a tumor marker was assessed. Data showed that 41 patients with epithelial ovarian cancer, the level of CA125 correlated well with tumor load as indicated by FIGO stage. However, no correlation was found between CA125 concentration and histopathological grade, also CA125 level didn't appear of any prognostic value [62].

Out of the eleven studies reviewed under the relationship between preoperative absolute serum CA125 levels and survival six studies were retrospective, two prospective and one convenience sample. Out of the six retrospective studies, four found a significant correlation between preoperative serum CA125 levels and survival. One study found CA125 as the strongest independent prognostic factor for survival. The two prospective studies did not find any significant correlation between preoperative serum CA125 level and survival. Finally, the convenience sample based study also recorded a significant correlation between preoperative serum CA125 levels and survival in ovarian cancer. The overall review of the literature in this section suggests a strong prognostic role of preoperative serum CA125 levels in ovarian cancer.

### Postoperative Absolute Serum CA125 and Ovarian Cancer Survival

Table 5 summarizes the epidemiologic studies on the association between absolute postoperative CA125 levels and survival in ovarian cancer. A study found that postoperative CA125 correlated to FIGO stage, tumor grade and overall survival [53]. Another retrospective analysis of 85 patients with elevated serum CA125 after surgery for ovarian cancer showed that the absolute CA125 serum levels were a poor guide to prognosis [63]. A study found that in patients without residual disease after primary surgery, histologic type, postoperative CA125 level with 35 U/mL as the cutoff value, and tumor grade were independent prognostic factors for survival. For those with residual tumor after primary surgery, histologic type, postoperative treatment, size of residual disease, and postoperative serum CA125 level with 65 U/mL as a cutoff were independent prognostic factors [59]. In another study evaluating 132 patients, postoperative CA125 was found the strongest independent prognostic factor for survival, as compared with histologic grade, FIGO stage, and diameter of residual tumor mass [60]. A study evaluated whether the pre- and postoperative determination of CA 125 improves the prognostic information at the time of primary operation. Elevated postoperative values resulted in poor prognosis. The study found a survival of 5% after 5 years in this group vs. 42% for patients with normal postoperative values. The best prognosis was found in patients with pre- and postoperative values lower than 65 U/ml [61].

**Table 5:** Relationship between postoperative absolute serum CA125 levels and survival in ovarian cancer

**First Author, Year, Study Place**	**Data Collection**	**Study Design**	**Sample Size**	**Groups being Compared**	**RR/HR, (95% CI), P-Value**	**Conclusion**	**Variables Adjusted for**
Osman N, 2008, Limerick [53]	Jan 2001 to Dec 2005	Retrospective study	68	≤ 500 U/ml, > 500 U/ml	NA, < 0.01	The postoperative CA125 correlated to FIGO stage, tumor grade and overall survival. Reduced survival was noted with increasing age at the time of surgery and bulk of the residual disease postoperatively.	Age, histology, FIGO stage, grade

Munstedt K, 1997, Germany [63]	1987 to 1994	Retrospective study	85	< 60 U/ml, > 60 U/ml, > 480 U/ml	NA	Serum CA125 levels within 4 weeks of operation were of no significant value as indicators of overall survival	Age, FIGO stage, histology, grades

Makar AP, 1992, Norway [59]	1983 to 1990	Prospective study	487	≤ 35 U/ml, 36-65 U/ml, > 65 U/ml	I-1.00, (Reference), II- 1.56 (0.82-2.80), NS, III- 2.40 (1.42-4.06), 0.0008	In patients without residual disease after primary surgery, histologic type, postoperative CA125 level with 35 U/mL as the cutoff value, and tumor grade were independent prognostic factors for survival	Size of residual disease, histology, FIGO stage, tumor grade

Sevelda P, 1989, Austria [60]	NA	Prospective study	132	≥ 35 U/ml, < 35 U/ml	NA, 0.0006	Postoperative CA125 was the strongest independent prognostic factor for survival	Histologic grade, FOGO stage, residual tumor

Moebus V, 1988, Germany [61]	NA	Convenience sample	165	≥ 65 U/ml, < 65 U/ml	NA, 0.001	Significantly longer survival was noted for patients with postoperative values below 65 U/ml than for those patients with postoperative values above 65 U/ml	NA

Out of the five studies reviewed under the relationship between postoperative absolute serum CA125 levels and survival, two were retrospective, two prospective and one convenience sample. Four studies [53,59-61] found postoperative serum CA125 levels as a strong independent prognostic factor for survival in ovarian cancer. Whereas, only one study found postoperative serum CA15 having no significant prognostic value [63].

### Serum CA125 Half-life and Ovarian Cancer Survival

Table 6 summarizes the epidemiologic studies on the association between serum CA125 half-life and survival in ovarian cancer. In one study nadir concentration, residual tumor volume and number of chemotherapy courses were found to be independent prognostic factors for DFS and OS. The CA125 group classification was found to be an independent prognostic factor only for DFS [64]. In another study, CA125 half-life, nadir CA125 and time to nadir were studied. Median (range) for CA125 kinetics were: 263 kU/l (5-52000 kU/l) before 1st course, 15.8 days (4.5-417.9 days) for CA125 half-life, 16 kU/l (3-2610 kU/l) for nadir and 85 days (0-361 days) for time to nadir. In Cox models, CA125 half-life, residual tumor, nadir concentration and stage were the most powerful prognostic factors for DFS and OS [38].

**Table 6:** Relationship between serum CA125 half life and survival in ovarian cancer

**First Author, Year, Study Place**	**Data Collection**	**Study Design**	**Sample Size**	**Groups being Compared**	**RR/HR, (95% CI), P-Value**	**Conclusion**	**Variables Adjusted for**
Riedinger JM, 2008, France [64]	1996 to 2000	Multicentric Retrospective study	130	Non-assessable, ≤ 14 days and mono-exponential decay, ≤ 14 days and bi-exponential decay, > 14 days	NA	The CA125 group classification was found to be an independent prognostic factor only for DFS	CA125 nadir, chemotherapy courses, residual tumor

Riedinger JM, 2006, France [38]	1988 to 1996	Multicentric Retrospective study	553	≤ 14 days, > 14 days	2.04 (1.58-2.63), < 0.0001	Among well-established prognostic factors in ovarian cancers, CA125 half-life and nadir concentration bear a strong and independent prognostic value	FIGO stage, residual tumor, age, CA125 nadir

Gadducci A, 2004, Italy [65]	1996 to 2002	Retrospective study	71	≤ 14 days, > 14 days	3.11 (1.22--7.98), 0.0181	Serum CA125 half-life was an independent prognostic factor for the chance of achieving a complete response to treatment as well as for progression-free survival and overall survival	Age, Histology FIGO stage, Residual disease, chemotherapy regimen, CA125 percentage reduction after the first cycle of chemotherapy

Colakovic S, 2000, Yugoslavia [51]	NA	Retrospective study	222	< 20 days, > 20 days	NA, 0.007	CA125 half life can divide patients into good and poor prognostic groups early during chemotherapy	Therapeutic response Karnofsky index, residual disease, tumor grade, CA125 kinetics

Munstedt K, 1997, Germany [63]	1987 to 1994	Retrospective study	85	< 20 days, > 20 days	0.6184	Serum CA125 half-life did not have any significant correlation with survival	Age, FIGO stage, Histology, grades

Gadducci A, 1995, Italy [40]	1986 to 1992	Multicentric Retrospective study	225	< 25 days, ≥ 25 days	2.13 (1.23-3.68), 0.0073	Serum CA125 half-life during early chemotherapy was an independent prognostic factor for both achievement of a pathological complete response and survival	FIGO stage, Tumor grade, size of residual disease, CA125 level

Rosman M, 1994, Connecticut [66]	June 1985 to July 1989	Retrospective study	51	≤ 12 days, > 12 days	3.6 (1.8-7.4), < 0.001	In those patients in whom residual small volume disease after primary surgery indicates a good prognosis, minimum CA125 and CA125 t1/2 during chemotherapy can further categorize patients into favourable and unfavourable prognostic groups	FIGO stage, tumor grade, residual disease, CA125

Yedema C A, 1993, Netherlands [52]	July 1984 to Dec 1990	Retrospective study	60	≤ 20 days, > 20 days	9.17 (1.49-56.3), 0.01	CA125 half-life provides an independent prognostic factor for survival in stage III-IV patients early in the course of therapy	Stage, histology, grade, tumor rest

Hogberg T, 1990, Sweden [67]	1984-1987	Prospective study	72	≤ 8 days, 8 - ≤ 12 days, 12 < - ≤ 16 days, > 16 days	NA, 0.003	The patients with a short serum CA125 half-life had a significantly better probability of survival	Age, histology FIGO stage, residual tumor, grade

Hunter VJ, 1990, Durham [68]	March 1984 to Jan 1989	Prospective study	54	≤ 20 days, > 20 days	NA, < 0.015	Overall survival was significantly greater in patients with a CA125 half life ≤ 20 days	NA

Hawkins RE, 1989, London [69]	NA	Prospective study	29	< 20 days, 20-40 days, > 40 days	3.7 (0.7-20.1), 0.001; 27.8 (4.0-193), 0.001	CA125 half life was independent prognostic indicator for survival	Residual tumor, stage, ascites

van der Burg ME, 1988, Netherlands [44]	Sept 1979 to Dec 1983	Consecutive case series	85	< 20 days, ≥ 20 days	NA	The half-life of CA125 appeared to be significantly and independently correlated with progression rate and progression-free survival	FIGO stage, histology, histological grade, postoperative tumor size

In a retrospective study the 25%, 50%, and 75% quartiles of serum CA125 half-life during early chemotherapy were 10, 14, and 20 days, respectively. Taking the value corresponding to the 50% quintile (i.e., 14 days) as cutoff limit, serum CA125 half-life was an independent prognostic factor for the chance of achieving a complete response to treatment as well as for progression-free survival and overall survival [65]. Another retrospective study found that the median survival times of patients with CA125 half-life < 20 days and > 20 days were 101+ and 18 months, respectively. In Cox analysis, independent prognostic variables for survival included therapeutic response, Karnofsky index, residual disease, tumor grade, CA125 half-life, and CA125 kinetics [51]. Another study showed that residual disease, serum CA125 half-life, and tumor grade retained a significant value in predicting survival in 225 patients with advanced epithelial ovarian cancer [40].

In another study, stage, residual disease, minimum CA 125, and CA 125 t1/2 individually were predictive of persistent disease or recurrence within 3 years of diagnosis with sensitivities of 97, 70, 34, and 49%, respectively, and specificities of 33, 83, 100, and 83%, respectively [66]. Another retrospective study found a significant positive correlation between survival and (a) stage III, (b) residual tumor ≤ 1 cm, (c) CA125 normalisation after three courses and (d) CA125 half-life ≤ 20 days. A CA125 half-life < or = 20 days vs > 20 days provides an independent prognostic factor for survival in stage III-IV patients early in the course of therapy [52].

A study found that patients with a serum CA125 half-life shorter than 16 days during induction chemotherapy had an estimated survival of 68% as compared with 18% in 49 patients with a CA125 half-life of more than 16 days [67]. In another study, CA125 half-life of less than 20 days was associated with prolonged overall survival. In those patients who eventually were found to be disease-free at surgical surveillance procedures, normalization of serum CA125 levels to less than 35 U/ml within 65 days of primary operation also suggested an improved survival [68]. A study found that CA125 half-life of less than 20 days, 20-40 days and greater than 40 days appeared to identify patients with a good, intermediate or poor prognosis, the two year actuarial survival being 76%, 48% and 0% respectively. The change of achieving a complete remission was 15% and 67% respectively for patients with a serum CA125 half-life of greater than 20 or less than 20 days [69]. In another study, patients with a half-life of 20 days and more had a 3.2 times higher progression rate and a significantly shorter median time to progression of only 11 months, as compared to 43 months for patients with a half-life of less than 20 days [44].

Out of the twelve studies reviewed under the relationship between serum CA125 half life and survival in women with ovarian cancer, eight studies were retrospective, one prospective and three consecutive case series. Eleven studies showed that serum CA125 half life was an independent prognostic indicator for survival.

### Nadir Serum CA125 and Ovarian Cancer Survival

Table 7 summarizes the epidemiologic studies on the association between nadir serum CA125 levels and survival in ovarian cancer. In one study, nadir concentration, residual tumor volume and number of chemotherapy courses were found to be independent prognostic factors for DFS and OS [64]. In another study nadir CA125 concentration and time to nadir were studied. Median (range) for CA125 kinetics were: 16 kU/l (3-2610 kU/l) for nadir and 85 days (0-361 days) for time to nadir. In Cox models, CA125 half-life, residual tumor, nadir concentration and stage were the most powerful prognostic factors for DFS and OS [38]. More recently, Crawford and Peace examined differences in nadir CA125 levels within the normal range as a prognostic factor for both overall and progression-free survival. Using arbitrarily defined groups of < 10 U/ml, 11-20 U/ml, and 21-30 U/ml, they demonstrated a statistically significant increase in overall survival only between patients with CA125 < 10 U/ml (median survival 2436 days) and those with CA125 > 11-20 U/ml or 21-30 (median survival 537 days for both groups). In a multivariate analysis, nadir remained highly significant [8]. In another study, in 223 patients with epithelial ovarian carcinoma the CA125 trend was the most significant variable followed by the FIGO stage. The initial CA125 level and nadir CA125 level, although significant when considered alone, were not significant independent variables [70].

**Table 7:** Relationship between nadir CA125 levels and survival in ovarian cancer

**First Author, Year, Study Place**	**Data Collection**	**Study Design**	**Sample Size**	**Groups being Compared**	**RR/HR, (95% CI), P-Value**	**Conclusion**	**Variables Adjusted for**
Riedinger JM, 2008, France [64]	1996 to 2000	Multicentric Retrospective study	130	≤ 20 kU/L, > 20 kU/L	4.18 (2.65-6.62), < 0.0001	Nadir concentration, residual tumor volume and number of chemotherapy courses were found to be independent prognostic factors for disease free survival and overall survival	Chemotherapy courses, residual tumor

Riedinger JM, 2006, France [38]	1988 to 1996	Multicentric retrospective study	631	≤ 20 kU/L, > 20 kU/L	1.65 (1.28-2.12), < 0.0001	CA125 half life and nadir concentration are powerful independent prognostic factors. CA125 nadir concentration and time to nadir all had a univariate prognostic value for disease free survival and overall survival	Age, FIGO stage, residual tumor, CA125 half life

Crawford SM, 2005, [8]	1988 to 2000	Retrospective study	79	≤ 10 U/ml, 11--20 U/ml, 21--30 U/ml	NA, < 0.001	The nadir group was the only predictor of overall survival that remained very highly significant (P < 0.001) after the effect of the other factors was allowed for	Type of chemotherapy, CA125 level at course three

Gard GB, 1994, Australia [70]	1985 to 1991	Retrospective study	223	> 35 U/ml, 15-35 U/ml, < 15 U/ml	Uni- 3.23 (2.62-3.99), < 0.0001 Multi-0.85 (0.54-1.33), 0.476	The initial CA125 level and nadir CA125 level, although significant when considered alone, were not significant independent variables	Tumor types, FIGO stage, residual disease, CA125

All four studies reviewed under the relationship between nadir CA125 levels and survival were retrospective. All studies found that nadir concentration was an independent prognostic factor for disease free survival and overall survival. Notably, one of these studies [70] reported that the initial CA125 level and nadir CA125 level were found significant when considered alone but were not significant as independent variables.

### Time to Reach CA125 Nadir and Ovarian Cancer Survival

Table 8 summarizes the epidemiologic studies on the association between time to reach CA125 nadir and survival in ovarian cancer.

**Table 8:** Relationship between time to reach nadir CA125 levels and survival in ovarian cancer

**First Author, Year, Study Place**	**Data Collection**	**Study Design**	**Sample Size**	**Groups being Compared**	**RR/HR, (95% CI), P-Value**	**Conclusion**	**Variables Adjusted for**
Riedinger J M, 2006, France [38]	1988 to 1996	Multicentric retrospective study	553	≤ 72 days, > 72 days	Univariate analysis 0.67 (0.63--0.71) < 0.0001	CA125 nadir concentration and time to nadir all had a univariate prognostic value for disease free survival and overall survival. In Cox models, nadir concentration was the most powerful prognostic factors for disease free survival	NA

Frasci G, 1996, Italy [71]	1985 to 1990	Consecutive case series	54	≤ 1 month, > 1 months	0.009	Survival of patients with advanced ovarian cancer could be accurately predicted by considering some pretreatment variables and time to CA125 normalization together, without performing second look laparotomy	Residual tumor burden after surgery, Eastern Cooperative Oncology Group (ECOG) performance status

In one study, nadir CA125 concentration and time to nadir were studied. In Cox models, CA125 half-life, residual tumor, nadir concentration and stage were the most powerful prognostic factors for DFS and OS. This study concluded that among well-established prognostic factors in ovarian cancers, CA125 half-life and nadir concentration bear a strong and independent prognostic value [38]. Another study evaluated the incorporation of CA125 normalization times into a prognostic model based on pretreatment variables in patients with ovarian carcinoma to determine if they could render second-look laparotomy (SLL) redundant. The time to normalization of CA125 serum levels (analyzed either as a continuous or as a two-category variable) had an independent prognostic role when included in the model. Patients with good prognostic pretreatment variables, and those with intermediate prognosis at the beginning of therapy who showed a quick normalization of CA125, had an 80% 5-year survival, compared with 16% 5-year survival in the remaining patients [71].

### CA125 Area under the Curve (AUC) and Ovarian Cancer Survival

A study evaluated the usefulness of CA125 normalized in time area under the curve (CA125 AUC) to signalise epithelial ovarian cancer relapse. Data from 111 patients were submitted to two different approaches based on CA125 AUC increase values to predict patient relapse. In Criterion A the best accuracy was achieved with a factor (F) of 1.25 (increment of 25% from the previous status), while in Criterion B the best accuracies were achieved with cut-offs of 25, 50, 75 and 100 IU/mL. The mean lead time to relapse achieved with Criterion A was 181 days, while with Criterion B they were, respectively, 131, 111, 63 and 11 days. Based on the results of this study it was concluded that conjugation and sequential application of both criteria in patient relapse detection should be highly advisable [72]. Another study investigated the usefulness of the CA125 area under the curve (AUC) as a new kinetic parameter for predicting overall survival. Patients with a complete response to primary chemotherapy had a mean CA125 AUC of 48.8, while patients with a partial response had a mean of 251.7 IU/ml*days, and patients with no response or disease progression had a mean of 316.5 IU/ml*days. The best CA125 AUC performance is in predicting patient complete response to chemotherapy with a cut-off of 100 IU/ml*days and an accuracy of 82% [73].

### Longitudinal Serum CA125 and Ovarian Cancer Survival

Table 9 summarizes the epidemiologic studies on the association between longitudinal CA125 levels and survival in ovarian cancer.

**Table 9:** Longitudinal studies on serum CA125 levels and survival in ovarian cancer

**First Author, Year, Study Place**	**Data Collection**	**Study Design**	**Sample Size**	**Groups being Compared**	**RR/HR, (95% CI), P-Value**	**Conclusion**	**Variables Adjusted for**
Riedinger JM, 2007, France [47]	1988 to 1996	Multicentric study	494	≥ 75% decrease, < 75% decrease or increase	Univariate- 1.92 (1.34--2.74), < 0.0001 3.08 (2.10--4.50), < 0.0001 Multivariate-0.90 (0.59--1.39), NS 0.97 (0.59--1.59), NS	The CA125 change after first course of CT was independent prognostic factors for both achievement of pathological complete response and overall survival.	Age, Histology FIGO stage, Residual tumor

Markman M, 2006, USA [37]	NA	Longitudinal study	291	I > 100, ≤ 35 u/ml II 36--99, ≤ 35 u/ml III > 100, 36--99 u/ml	I - 3.0 (1.7--8.6), 0.0001 II - 1.6 (0.9--2.8), 0.09 III-1.8 (1.0--3.5), 0.07	While pretreatment CA125 values did not correlate with survival, the concentration of CA125 8 weeks after initiation of therapy was a powerful independent prognostic factor.	Age, performance status, disease stage, measurable disease, treatment, tumor necrosis, stromal sclerosis

The first study was a multicentric study [47], carried out to assess the prognostic value of the CA125 change after the first and the second courses of induction chemotherapy. CA125 determination of all patients was carried out before each cycle of chemotherapy (on average each 3 weeks). The data from this study showed that early CA125 change induced by the first chemotherapy courses was strongly correlated both with the probability of achieving pathological complete response after chemotherapy and with survival duration. In another study [37] the relationship between survival and early changes in the serum level of the CA125 antigen in patients with advanced ovarian cancer was assessed. While pretreatment CA125 values did not correlate with survival, the concentration of CA125 8 weeks after initiation of therapy was a powerful independent prognostic factor.

## Discussion

Epithelial ovarian cancer is fairly common with high rates in Scandinavia, intermediate rates in Western Europe and North America and low rates in the developing countries and in Japan. The 5-year survival rate is less than 40% [74]. A variety of biomarkers have been developed to monitor growth of ovarian cancer and to detect disease at an early interval [17]. Amongst them CA125 has been the most extensively studied and clinically utilized serum tumor marker. As a clinical tool, prognostic markers like CA125 may potentially help individualizing treatment within subgroups of patients. Serum levels of CA125 are used to monitor responses to chemotherapy, relapse, and disease progression in ovarian cancer patients [31,75]. Levels of CA125 can be elevated in the serum before clinical development of primary and recurrent ovarian carcinoma [76]. It is now widely accepted that the tumor marker CA125 is a predictive and prognostic factor in CA125 positive ovarian cancers.

In the diagnosis of the disease, prognosis and monitoring of treatment CA125 serum concentration has been established as a tool of great importance. An important characteristic of CA125 is the ability to reflect changes in tumor mass during chemotherapy or in the follow-up period after completion of therapy [77]. The postoperative serum CA125 level is an independent prognostic factor in patients with invasive ovarian cancer [59], and CA125 tumor marker half-life (t1/2) and tumor marker doubling time (DT) are often used as kinetic parameters for the evaluation of clinical response and follow-up of patients with ovarian cancer [78]. Serum CA125 half-life during early chemotherapy is an independent prognostic factor for both the achievement of a pathologically complete response and the survival of patients with advanced epithelial ovarian cancer [40], and several studies report that the greatest difference in progression rate was found at a t1/2 of 20 days [44,51,68,69,73]. While CA125 levels at the time of diagnosis are of limited prognostic significance, CA125 measurements are now performed almost routinely during the course of chemotherapy. Decreasing levels of CA125 after cytoreductive surgery and during initial chemotherapy courses have been used as an indicator of clinical outcome [5]. Despite general acceptance that CA125 is a prognostic marker, it remains unclear as to how accurately serial CA125 measurements can predict long-term overall survival in patients with advanced ovarian cancer [79].

The present review summarizes findings from studies assessing the relationship between serum CA125 levels and survival in ovarian cancer. All studies that evaluated the relationship between prechemotherapy, post chemotherapy, during chemotherapy, preoperative, postoperative absolute serum CA125 levels, CA125 half-life, nadir CA125 levels, time to reach nadir level and survival in ovarian cancer were included in this review. Also studies evaluating the relationship between a new kinetic parameter AUC (Area under the Curve) as well as the longitudinal studies on CA125 and survival in ovarian cancer have also been reviewed.

Most studies reviewed had retrospective study design. To date, only four prospective investigations have been conducted, of which two [41,62] had very few cases (n < 55), indicating limited power to detect associations. Prospective studies are the preferred study design, as they are less susceptible to various types of biases and provide the best risk estimates. Thus, further prospective investigations are needed for clearly understanding the relationship between serum CA125 levels and survival in ovarian cancer. Also most of the studies reviewed lack information in the relative risk estimates associated with different CA125 levels.

There have been earlier attempts by Hogdall [19] and Gadducci et al. [80] to review the literature on CA125 and ovarian cancer survival. Review by Hogdall addressed recently reported progress in CA125 as a prognostic marker in patients with ovarian cancer. It primarily focused on recent publications on the topic and excluded some studies on CA125 levels during chemotherapy, time to reach nadir level and CA125 AUC levels. Another very informative review by Gadducci et al. focused on all serum and tissue biomarkers in ovarian cancer and not just CA125. Also, it failed to include all available studies on CA125 possible because the focus of the paper was not just CA125. The present review we believe provides a very comprehensive evaluation on the existing literature on the prognostic role of CA125 in ovarian cancer.

We conclude that although the results from different studies are sometimes contradictory, serum CA125 level is a strong prognostic factor for overall survival and progression free survival in ovarian cancer. There is an inverse relationship between serum CA125 levels and survival in ovarian cancer. A decreasing level generally indicates a positive response to cancer therapy while an increasing level indicates tumor recurrence and poor survival. Future research on this subject should focus on prospective longitudinal studies evaluating the impact of changes in serum CA125 levels on survival.

## Competing interests

The authors declare that they have no competing interests.

## Authors' contributions

DG and CGL participated in concept, design, data collection, data interpretation and writing. Both authors read and approved the final manuscript.
